# Where to Draw the LINE—Are Retrotransposable Elements Here to Stay?

**DOI:** 10.3390/cancers15164119

**Published:** 2023-08-16

**Authors:** Christopher J. Bergin, Amanda Mendes da Silva, Yannick D. Benoit

**Affiliations:** 1 Department of Cellular and Molecular Medicine, University of Ottawa, Ottawa, ON K1H 8M5, Canada; cberg040@uottawa.ca (C.J.B.); amend079@uottawa.ca (A.M.d.S.); 2 School of Pharmaceutical Sciences, University of Ottawa, Ottawa, ON K1H 8M5, Canada

**Keywords:** colorectal cancer, epigenetics, LINE1, methylation, retrotransposons, viral mimicry

## Abstract

**Simple Summary:**

Colorectal cancer incidence has increased in young adults over the last decade. Such a shift in epidemiology suggests increased environmental risk factors play a prominent role in the onset of the disease. DNA methylation is one of several aspects of the epigenome that are frequently mutated in cancer and that function in the silencing of endogenous viral repeats, also known as transposable elements. Here we discuss the findings from a recent mapping effort of Long Interspersed Nuclear Elements 1 (LINE1) transposition events over a lifetime in the colonic epithelium, which revealed increased levels of transposition in human colorectal tumors. These findings shed light on LINE1 retrotransposition in colorectal tumors and how it may be crucial in developing future therapeutic avenues.

**Abstract:**

The frequency of somatic retrotranspositions of Long Interspersed Nuclear Elements 1 (LINE1) over a lifetime in healthy colonic epithelium and colorectal tumors has recently been reported. Indicative of a cell type-specific effect, LINE1 sequences in colonic epithelium showed lower levels of DNA methylation compared to other cell types examined in the study. Consistent with a role for DNA methylation in transposon silencing, the decreases in DNA methylation observed at LINE1 elements in colonic epithelium were accompanied by increases in LINE1 mRNA levels. In human primary colorectal tumors, LINE1 retrotransposition frequency was tenfold higher than in normal colonic tissues, with insertions potentially altering genomic stability and cellular functions. Here, we discuss the discoveries made by Nam and colleagues, emphasizing the intestinal-specific methylation signature regulating the LINE1 lifecycle and how this new information could shape future drug discovery endeavors against colorectal cancer.

## 1. Main Text

With the second-heaviest toll of cancer deaths worldwide, colorectal cancer (CRC) undoubtedly represents a dire health concern [1]. Once recognized as an aging disease, the incidence of CRC has substantially increased in young adults over the last decade [2]. Akin to other pathologies of the lower digestive tract, such as inflammatory bowel diseases (IBD), this shift observed in CRC epidemiology points toward potential environmental causes rather than an increased frequency of inherited genetic susceptibilities. The parallel between IBD and CRC is interesting since microbiota dysbiosis was linked to both conditions via the induction of genomic hypomethylation in the colonic epithelium [3]. Dysregulation of DNA methylation has been extensively documented in CRC and confirmed as a key phenomenon contributing to epigenetic reprogramming in oncogenic transformation [4,5].

**Figure 1:**
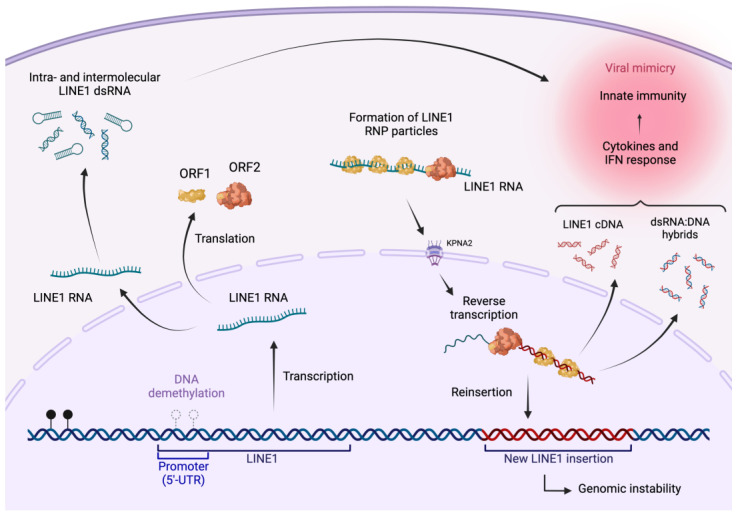
The LINE1 lifecycle. LINE1 retrotransposition is a highly regulated, multi-step process that allows for the mobility and insertion of long interspersed nuclear elements in the genome. Following demethylation, LINE1 elements transcription is driven by a promoter located in their 5′ untranslated region (5′-UTR). Upon modifications stabilizing the LINE1 transcripts, LINE1 open reading frames are translated in the cytoplasm. LINE1 open reading frames encode two distinct proteins: ORF2, which possesses endonuclease and reverse transcriptase activities, and ORF1, which functions as a nucleic acid chaperone responsible for ribonucleoprotein particle (RNP) assembly. Both proteins are critical for the association of LINE1 RNA and the formation of a LINE1-RNP intermediate complex. This complex is imported into the nucleus either passively during cell division or actively through interactions with nucleoporin proteins (e.g., KPNA2). Here, the reverse transcriptase and endonuclease activities of ORF2 synthesize a complementary DNA strand and integrate it into a new genomic location. LINE1-derived double-stranded RNA (dsRNA) generated by inter or intramolecular complementarity, cDNA, and LINE1 RNA:DNA hybrids in the cytoplasm can trigger an antiviral-like innate immune response. Due to truncations, internal rearrangements, and mutations frequently occurring through the lifecycle, most newly integrated LINE1 copies are not competent for further retrotransposition. Created with BioRender.com (accessed on 10 August 2023).

Epigenetic modifications, such as histone and DNA methylation, are closely associated with the activation state of genomic sequences of ancestral viral origin, termed transposable elements (TEs). The Long Interspersed Nuclear Elements 1 (LINE1) is a family of TEs that exist as repetitive elements that contribute to approximately 17% of the human genome [6]. The LINE1 family is an autonomous type of TEs, driving the mobility of other classes of retrotransposons, such as SINE and ALU elements [6]. Normally, LINE1 elements are silenced by DNA methylation, with DNA demethylation resulting in transcription, reverse transcription, and transpositions (Figure 1), a process that can lead to genomic alterations [6]. Advances in DNA sequencing now make it possible to map TE insertions in somatic tissues and thus understand how they contribute to pathogenic processes like tumorigenesis. In a recent publication, Nam et al. mapped somatic LINE1 retrotranspositions (soL1Rs) over their lifetime in the colonic epithelium and colorectal tumors [7]. Using a multi-omics approach on clonal organoid cultures established from human colonic adenoma, carcinoma, and healthy crypts, the authors explored the dynamics of soL1Rs in physiological and pathological contexts of the digestive epithelium.

Strikingly, soL1Rs are more abundant in healthy colonic epithelium than other tested tissues (e.g., skin fibroblasts and hematopoietic progenitors), and several recurrent insertions originate from early embryonic events. Retrotransposition-competent LINE1 sequences in normal colonic cells show a higher demethylation frequency than other studied cell types. As expected, this phenomenon is associated with increased LINE1 transcription. Akin to the clock-like mutational acquisition observed for other genomic sequence variations [8], Nam et al. reported an age-dependent increase in the soL1R burden through adulthood [7]. However, according to the authors, the demethylation profile of retrotransposition-competent LINE1s in the colonic epithelium is dictated through pre-gastrulation epigenome reprogramming. Thus, stochastic loss of methylation at such TEs would be unlikely to occur during aging. Instead, the authors suggest an incomplete re-methylation process that would take place following early embryonic stages at the promoter of retrotransposition-competent LINE1s within the colonic epithelium lineage. The insufficient LINE1 re-methylation pattern was not observed in other tissues, supporting its intestine-specific character.

Based on the analysis by Nam et al. regarding the location-specific insertion of soL1Rs, LINE1s are not prone to integrate into protein-coding regions in normal cells, which could impact homeostatic functions. Instead, it is suggested that normal cells harboring soL1Rs in protein-coding loci could be negatively selected in transit-amplifying populations [7]. As observed in other tissues, most LINE1 transcripts in normal colonic cells are unproductive, i.e., they do not result in genomic insertions [7,9]. Hence, despite the continuous exposure to LINE1 fragments in colonic epithelium over a lifetime, the insertion event frequency per individual cell remains low. Still, when put into perspective over a lifetime, the calculated frequency of LINE1 insertions suggests a healthy 60-year-old individual, with approximately 10 million colonic crypts, would have experienced nearly 20 million soL1Rs in the colon. While most of these events will remain functionally silent, some soL1Rs might contribute to intestinal pathologies such as CRC [6].

Observations by Nam et al. in primary CRC samples unveiled a soL1R burden tenfold higher than in normal colonic tissues and with significantly longer genomic insertions (average of 1031 bp for solo LINE1 sequences in tumors versus 453 bp in normal) [7]. Specifically, soL1R mapping across intestinal polypogenesis showed increased LINE1 insertion rates as a cell acquires driver mutations toward a carcinoma trajectory. Interestingly, CRC appears as an accommodating context for LINE1 retrotransposition, which is not correlated to other aspects of genomic instability in cancer, such as TP53 inactivating mutations and mismatch repair deficiency.

While the findings reported by Nam et al. represent a game-changer for understanding LINE1 mobilization in the digestive epithelium, some questions remain about the intestinal-specific demethylation status of the LINE1 promoter and the potential contribution of TEs to colorectal cancer [7]. An element of the answer could reside in the chronic exposure of the colonic epithelium to commensal microbiota, which influences DNA methylome via the TET-dependent demethylation machinery [10]. Moreover, it is unclear why neoplastic lesions are auspicious for LINE1 retrotransposition compared to healthy tissues and whether these events foster tumor initiation or intratumor heterogeneity. Interestingly, 97% of the soL1Rs detected by Nam et al. in 19 primary colorectal tumor samples were identified as clonal events shared across all tumor cells [7]. This suggests that such clonal soL1Rs occur early in the CRC tumorigenesis cascade due to the epigenetic reprogramming characterizing tumor initiation [5]. From a therapeutic standpoint, targeting early clonal events in tumors was proposed as an attractive strategy for anticancer drug development and demonstrated interesting disease progression-free survival benefits in clinical trials [11].

A relationship between the epigenome and TEs in anti-tumor immunity is gaining significant attention in melanoma and other types of tumors. For example, the chromatin-associated factor KDM5B has been implicated in the immune evasion of melanoma, where its depletion triggers a robust immune response due to reduced histone-3 lysine-9 (H3K9) methylation and de-repression of endogenous TEs [12]. Similarly, inhibiting the H3K9 methyltransferase crucial to CRC initiation decreased H3K9me2 and DNA methylation and increased transcription of LINE1 elements [13]. Enhanced TE expression upon low-dose treatments with demethylating agents has previously been associated with viral mimicry in CRC tumor-initiating cells. In such contexts, double-stranded RNA species (dsRNAs) derived from TE transcripts, reverse-transcribed LINE1 cDNAs, and RNA:DNA hybrids induce a type-I interferon-dependent innate antiviral response (Figure 1) [14]. Interestingly, independent studies showed that reverse transcriptase (RT) inhibitors downregulate viral mimicry by decreasing cytoplasmic double-stranded nucleic acid species generated by LINE1 ORF2 RT activity [12,15].

Despite the reported benefits from TE RNA expression, with viral mimicry stimulating antitumor immune response [14], different groups are currently developing small molecules blocking TE-encoded reverse transcriptases based on classical nucleoside [16] or non-nucleoside structures [17]. While such inhibitors may help to decrease high rates of soL1R in CRC, they could also downplay innate immune recognition and elimination of early neoplastic lesions, a concept recently dubbed the “fire alarm” hypothesis [18]. In this regard, Hepatitis B patients from a large cohort study (>35,000 individuals) undergoing long-term RT inhibitor treatments showed increased CRC incidence compared to untreated subjects [19]. It would be interesting to determine whether treated patients were more prone to immune evasion by early CRC lesions due to reduced production of LINE1 ORF2-dependent double-stranded nucleic acid species. Besides the LINE1-encoded endogenous reverse transcriptase ORF2, other aspects of the LINE1 lifecycle could be considered as putative drug targets, maintaining the accumulation of cytoplasmic double-stranded nucleic acid species and inducing viral mimicry while blocking retrotransposition and genomic plasticity (Figure 1).

Another aspect to consider vis-à-vis the findings reported by Nam et al. is the relationship between LINE1 RNA expression and aging, where de-repressed LINE1 elements were associated with cellular senescence and age-related inflammation [7,15,20]. Specifically, antisense oligonucleotides targeting LINE1 transcripts in human and mouse progeria models reversed the activation of pathways promoting aging, the inflammatory response, the innate immune response, and DNA damage [20]. Together with the rapid turnover of the epithelial sheath, the high levels of LINE1 demethylation and expression in the colonic epithelium compared to other systems might represent a critical factor in accelerating the aging of the colorectal mucosae. Thus, there could be a compromise between slower aging and reduced mucosal inflammation versus an efficient innate immune response against early neoplastic lesions, depending on the LINE1 activation state in the colonic epithelium.

## 2. Conclusions

Considering the tools available, in development, or proposed to target LINE1 retrotransposons at different stages of their lifecycle, it is still challenging to determine the definitive impact of LINE1 reactivation in CRC patients. Do LINE1s and other classes of TEs represent a driving force for CRC tumor evolution, contributing to intratumor heterogeneity, and must they be blocked at all costs? Or should LINE1 epigenetic de-repression and active transcription be regarded as a boon to be maintained and even harnessed as a safeguard promoting an antitumor immune response in CRC patients? The best of both worlds should be considered by developing combination therapies targeting TEs genomic integration while promoting their epigenetic de-repression and associated viral mimicry.
